# Long non‐coding RNAs in development and disease: conservation to mechanisms

**DOI:** 10.1002/path.5405

**Published:** 2020-03-16

**Authors:** Ioannis Tsagakis, Katerina Douka, Isabel Birds, Julie L Aspden

**Affiliations:** ^1^ School of Molecular and Cellular Biology, Faculty of Biological Sciences University of Leeds Leeds UK; ^2^ LeedsOmics University of Leeds Leeds UK

**Keywords:** long non‐coding RNA, long intergenic non‐coding RNA, anti‐sense lncRNAs, translation, conservation, stem cells, development, X chromosome inactivation, neurodegenerative disease, cancer, diabetes

## Abstract

Our genomes contain the blueprint of what makes us human and many indications as to why we develop disease. Until the last 10 years, most studies had focussed on protein‐coding genes, more specifically DNA sequences coding for proteins. However, this represents less than 5% of our genomes. The other 95% is referred to as the ‘dark matter’ of our genomes, our understanding of which is extremely limited. Part of this ‘dark matter’ includes regions that give rise to RNAs that do not code for proteins. A subset of these non‐coding RNAs are long non‐coding RNAs (lncRNAs), which in particular are beginning to be dissected and their importance to human health revealed. To improve our understanding and treatment of disease it is vital that we understand the molecular and cellular function of lncRNAs, and how their misregulation can contribute to disease. It is not yet clear what proportion of lncRNAs is actually functional; conservation during evolution is being used to understand the biological importance of lncRNA. Here, we present key themes within the field of lncRNAs, emphasising the importance of their roles in both the nucleus and the cytoplasm of cells, as well as patterns in their modes of action. We discuss their potential functions in development and disease using examples where we have the greatest understanding. Finally, we emphasise why lncRNAs can serve as biomarkers and discuss their emerging potential for therapy. © 2020 The Authors. *The Journal of Pathology* published by John Wiley & Sons Ltd on behalf of Pathological Society of Great Britain and Ireland.

## Introduction

Only 4% of the human genome codes for proteins, corresponding to ~20 000 protein‐coding genes, whereas ~85% of the genome can be transcribed into RNA. These additional transcriptional events represent part of the ‘dark matter’ of our genome. If these RNAs do not code for proteins, what do they do? Until relatively recently, most research focused on understanding the function and deregulation during disease of the 4% of the genome that codes for proteins. If we are truly to understand the genetic causes of disease, we need to look outside protein‐coding sequences, particularly at regions of non‐coding transcription. These regions produce a range of types and sizes of non‐coding RNAs, the most numerous of which are the long non‐coding RNAs (lncRNAs). Our understanding of lncRNAs has been transformed in the last 10 years. Nonetheless, relatively few lncRNAs have been characterised in detail and even fewer have had their functions characterised. Many lncRNAs have been found to be associated with a range of human diseases, but our understanding remains limited on exactly how these lncRNAs contribute to disease.

## What are lncRNAs?

LncRNAs are RNAs of >200 nucleotides (nt) in length that are not thought to code for proteins. Although our appreciation and understanding of lncRNA function and importance has exploded in the last decade, the first lncRNAs were discovered in the 1990s: BC200, H19 1, and Xist 2. In the post‐genomic era, extensive and deep RNA‐Seq has revealed the existence of huge numbers of novel RNA transcripts, including lncRNAs. Many of these novel transcripts are low in abundance and so were not previously identified. Several consortia have been responsible for sequencing RNA from a variety of tissues, cell types, organisms, and disease states, and we now have a much more precise view of which RNA transcripts are expressed, and when and where (GENCODE 3, GTEX 4, FANTOM 5).

**Figure 1:**
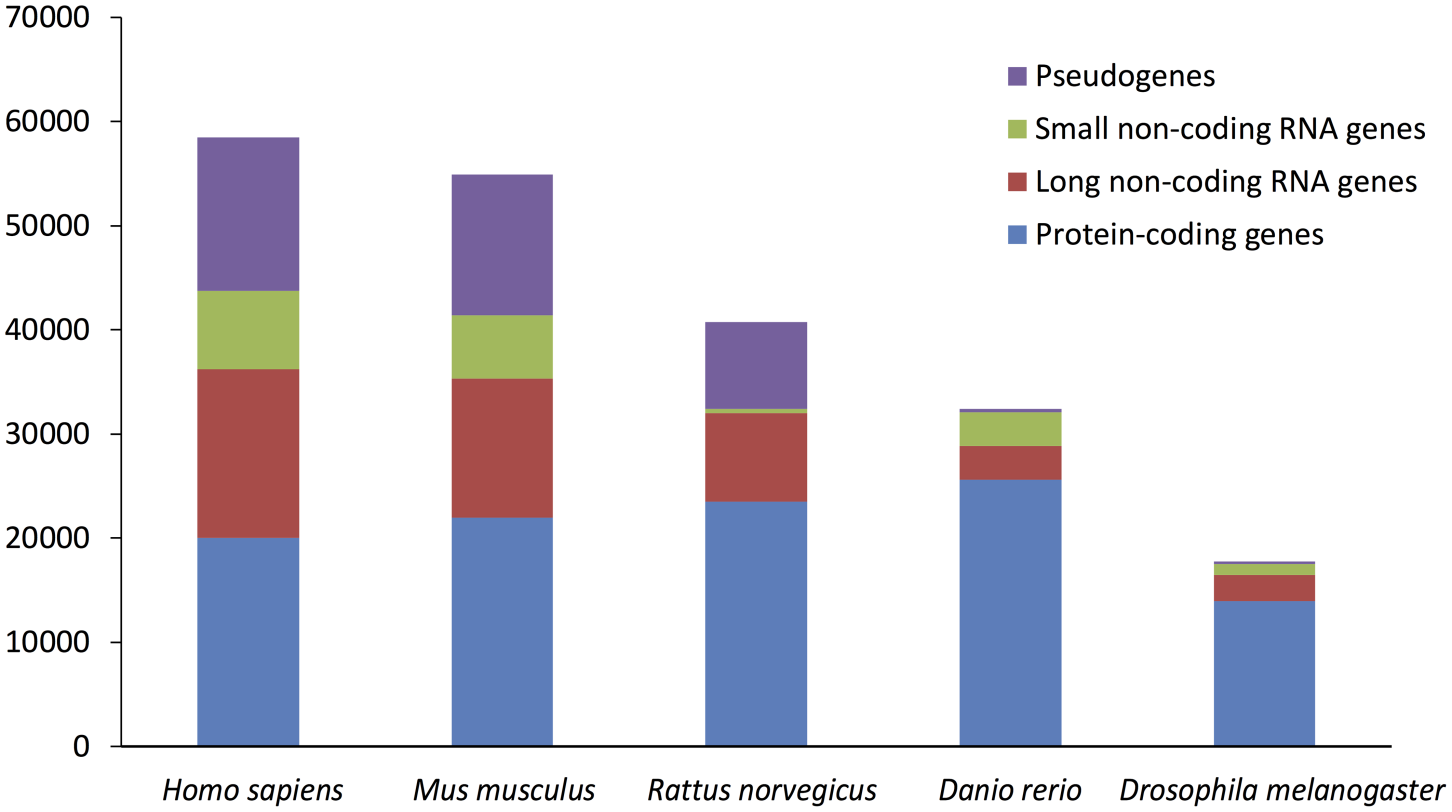
Numbers of different types of genes in humans and selected other eukaryotes. Data from *Homo sapiens*: GENCODE Release (version 30) 3, *Mus musculus* : GENCODE Release (version M21) 3, *Rattus norvegicus* : Ensembl RGSC assembly (v6.0) 111, *Danio rerio*: Ensembl (GRCz11) 111, *Drosophila melanogaster* : FlyBase (FB2019_02 R6.27) 112.

LncRNA genes are annotated as such because their RNAs do not contain large open reading frames that encode protein products (>100 amino acids). Of interest, although the number of protein‐coding genes does not substantially vary between *Drosophila* and human, the number of lncRNA genes does (Figure 1). Therefore, lncRNAs could represent part of the regulation system that enables higher eukaryotes to be more complex.

The lncRNA field now represents one of the most exciting and fast‐moving fields in biology. Although our understanding of lncRNAs has increased, a key question remains: how many lncRNAs are functional, or are the majority of lncRNAs the result of spurious transcription events? Experimental evidence suggests that >80% of lncRNAs possess biochemical activity such as protein binding 6. However, the importance or function of most of these lncRNA–protein interactions has yet to be validated. Although the function of many lncRNAs remains elusive, many have been shown to be associated with human health and disease. A key avenue going forward will be to dissect how lncRNAs that contribute to disease do so mechanistically.

## Categories of lncRNA

The molecular nature of lncRNAs varies and has the potential to influence their function and localisation. Approximately 50% of lncRNAs possess a polyA tail and 98% of human lncRNAs are spliced 7, similar to mRNAs. Many lncRNAs also possess m^7^G caps. These mRNA‐like features contribute to the ability of many lncRNAs to exit the nucleus and enter into pathways in which mRNAs take part. The majority of research over the last 10 years has focused on those lncRNAs that remain in the nucleus, but sequencing RNAs in the cytoplasm has revealed that many lncRNAs are also present in the cytoplasm. The relative abundance of an lncRNA between the nucleus and cytoplasm can help reveal function and interacting partners. Although many similarities exist between lncRNAs and mRNAs, a key molecular difference is their cross‐species conservation; lncRNAs are not well conserved.

**Figure 2:**
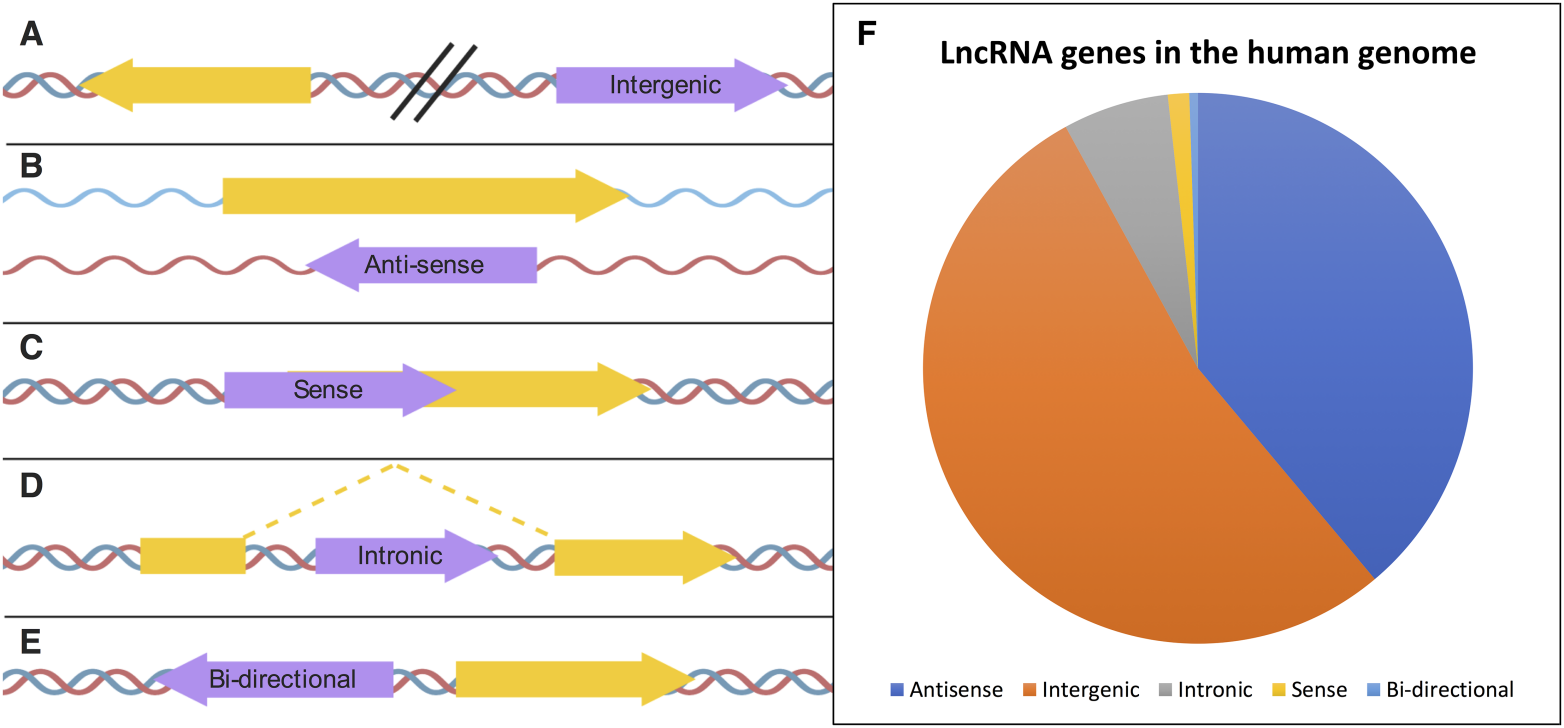
Categories of lncRNA. Types of lncRNAs based on their genomic position, orientation, and relative location to nearby protein‐coding genes. (A) intergenic, (B) anti‐sense, (C) sense, (D) intronic, and (E) bi‐directional. lncRNA genes are marked in purple and protein‐coding sequences in yellow. (F) Proportion of lncRNAs present in the human genome by location. Annotation from Gencode January 2019 (release 29, GRch38) 3. Created using BioRender.

LncRNAs can be categorised in several different ways, most simply by their genomic location and structure. These are not mutually exclusive categories, however. This method of categorisation can suggest potential lncRNA function but does not infer mechanism of action. **Intergenic** lncRNAs (lincRNAs) are those lncRNAs that do not overlap with any other genes (protein‐coding or non‐coding) and are >1 kb away from neighbouring genes (Figure 2A). These are the most straightforward lncRNAs to which to assign function because genomic mutations can be unequivocally assigned [e.g. clustered regularly interspaced short palindromic repeats (CRISPR)]. Many lncRNAs overlap other genes and can either be in the sense or anti‐sense orientation to those other genes. **Anti‐sense** lncRNAs inherently possess sequence elements that will base pair to other RNAs, that is, mRNAs from protein‐coding genes they are anti‐sense to (Figure 2B). **Sense** lncRNAs are located within other genes, but in the sense direction (Figure 2C). lncRNAs produced from within the introns of other genes are termed **intronic** lncRNAs (Figure 2D). The final group are lncRNA genes, which are transcribed from the same region in the genome as another gene, but in the opposite direction: **bi‐directional** lncRNAs (Figure 2E). Intergenic and anti‐sense are by far the most common types of lncRNAs in humans (Figure 2F).

**Figure 3:**
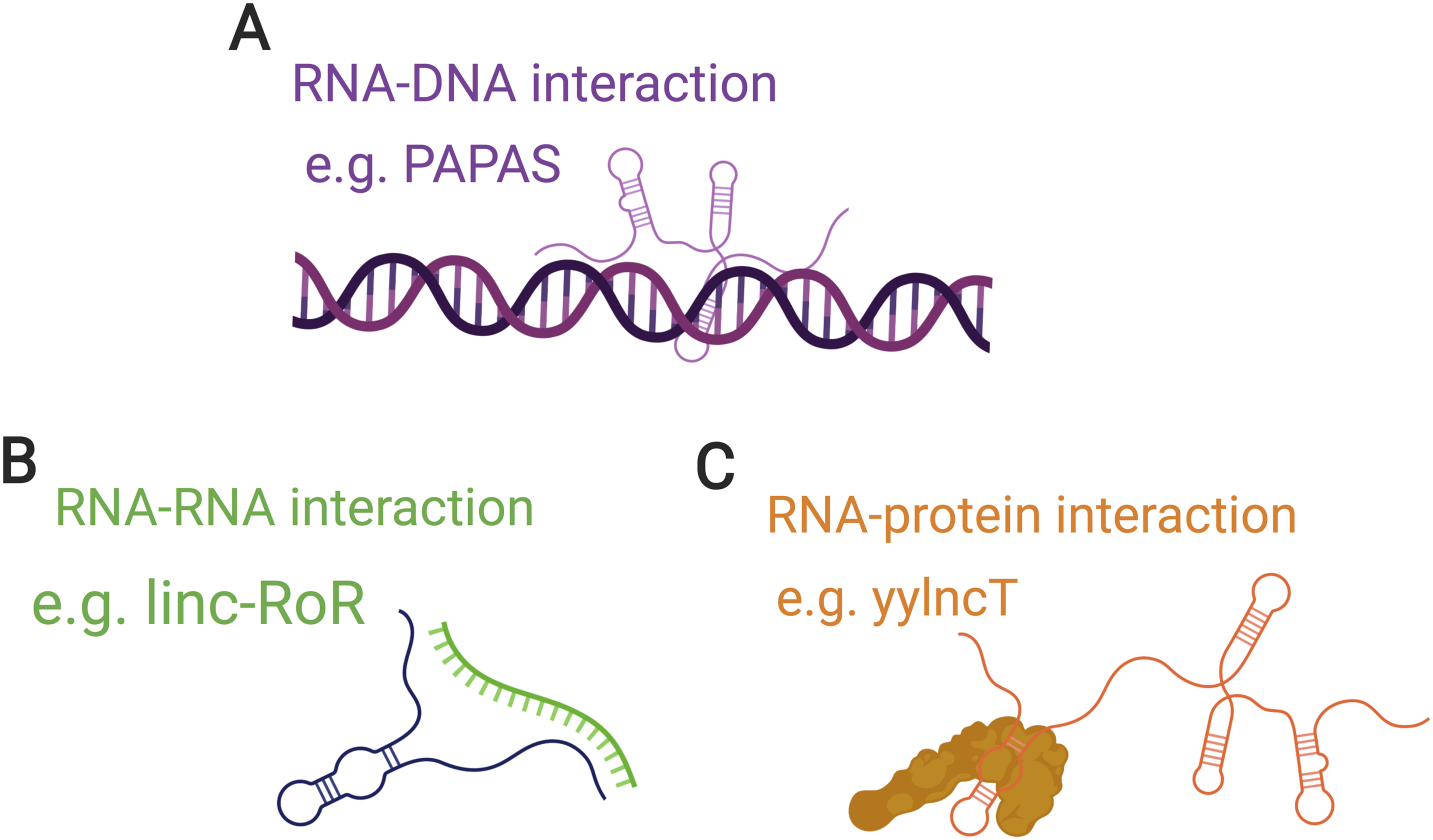
The principle mechanisms governing lncRNA interactions. lncRNAs have been found to interact with (A) DNA, via Hoogstein bonding to form triple helical structures, (B) RNA, *via* Watson–Crick–Franklin (hydrogen) base‐pairing, or (C) proteins. These interactions underlie all effector functions elicited by lncRNAs studied to date. Created using BioRender.

The link between lncRNA categories and potential function arises from the lncRNA's genomic location relative to potential target genes. The interaction of lncRNAs and target genes could take place at the site of transcription or somewhere else in the cell. Anti‐sense lncRNAs have the potential to regulate their anti‐sense genes at the point of transcription, acting in *cis*. Equally, lncRNAs can be transported to other locations in the cell, termed *trans*‐acting lncRNAs (see the glossary for definitions). On the simplest level, this might also be at the point of transcription of the target gene, which is elsewhere in the genome. The ability of lncRNAs to act in *trans* comes from their ability to base‐pair specifically with other RNAs and DNA, as well as to bind to proteins (Figure 3). These lncRNA complexes can then play roles in a number of gene expression processes and fall into several key mechanistic categories. lncRNAs can act as **scaffolds**, providing a site for other interactions. More generally, they can act to recruit protein complexes, which can be based on their sequence specificity. Forming lncRNA–protein complexes is an essential aspect for many lncRNA functions characterised so far. Some of these interactions can act as **decoys**, preventing proteins accessing other RNAs (RBP decoy). Alternatively, lncRNAs can act through specific base‐pairing with other RNAs, such as microRNAs (miRNAs). This can act to **sponge** miRNAs from other binding events. lncRNA–mRNA interactions can also act to regulate mRNA levels by increasing or decreasing their stability.

One of the most controversial areas of lncRNA biology is whether they are functional at all. Although functions have been characterised for some lncRNAs, they remain the minority. Much evidence suggests that the majority of lncRNAs are not functional 8. Some lncRNAs are thought only to possess activity as a result of their transcription; the lncRNA molecules produced from these transcription events do not have functions.

## Conservation and evolution of lncRNAs

Traditionally, evolutionary conservation has been used as a proxy for functionality. However, when we compare lncRNAs to canonical protein‐coding genes, they are found to be poorly conserved at the sequence level. lncRNAs also often lack orthologues in other species, and upon comparison with protein‐coding sequences or mRNA untranslated regions (UTRs), lncRNA exons are found to evolve at a faster rate 9, 10. This provides evidence that lncRNA genes are ‘junk DNA’, and has led to the hypothesis that lncRNA transcripts are the products of pervasive transcription, a common phenomenon often dismissed as ‘biological noise’.

Despite this, lncRNAs are in fact under stronger selective pressure than neutrally evolving sequences such as introns 9, 11, and their apparent lack of sequence conservation cannot be taken at face value. Many of the mechanisms of action of lncRNAs have low sequence constraints. For example, interacting with and sequestering RNA‐binding proteins (RBPs) requires sequence conservation over only small portions of the total sequence, in the range of 10 nt.

Generally, lncRNA genes are also small relative to protein‐coding genes, and this shortening of potential areas of conserved sequence reduces the effectiveness of standard bioinformatic detection methods. Solving this issue is an ever‐growing area of research and includes the adaptation of existing methods and the development of new ones, for example, methods that focus on the promoter regions of lncRNAs 12. Within vertebrates, thousands of homologues for human lincRNAs have now been found with shared expression patterns, despite sharing very short patches of sequence conservation 13. This opens the possibility that far more lncRNAs are in fact functional.

LncRNAs are also found to be syntenically conserved across multiple species. The human lncRNA CHASERR (CHD2 adjacent, suppressive regulatory RNA) is found upstream of *Chd2* (chromodomain helicase DNA binding protein 2) in both mice and humans and exhibits further conservation throughout the vertebrate lineage (Table 1). This has provided an ideal model to investigate CHASERR as a potential target to control levels of Chd2, a protein linked to human neuronal diseases ranging from epilepsy to neurodevelopmental delay 14, 15.

**Table 1:** Summary table of described example lncRNAs with subcellular localisation, type, function, conservation, and disease relevance

LncRNA	Subcellular localisation	Type	Function	Conservation status	Disease	References
*CHASERR/Chaserr*	Nuclear	Intergenic	Maintains Chd2 expression levels.	Sequentially and syntenically conserved between humans and mice. Extron–intron structure and some sequence conserved across vertebrates.	Chd2 is implicated in neurological disease.	14
*LINC00261*	Nuclear	Intergenic	Negative regulator of cell growth.	Syntenically homologous from humans to sea urchins. Some sequence homology in first exon.	Downregulated in multiple cancers, including endometrial and gastric.	13, 16
*Cerox1* (Figure 6B)	Cytoplasmic	Intergenic, bi‐directional	miRNA decoy, regulates abundance of mitochondrial complex 1 transcript.	Sequentially and syntenically conserved between humans and mice. Sequential conservation of exon 2 across mammals.	May have links to neurological diseases.	18
*Uchl1‐AS1* (Figures 4 and 6C)	Nuclear/Cytoplasmic	Anti‐sense	Translation regulation (lncRNA–mRNA interaction).	Conserved between humans and mice.	Downregulated in a neurochemical model of Parkinson's disease.	78
*PAPAS* (Figures 3A and 5A)	Nuclear (Nucleolus)	Anti‐sense	Represses rRNA synthesis at elevated temperatures.	Not examined	Hepatocellular carcinoma, triple negative breast cancer and aging.	96, 97
*XIST* (Figure 4)	Nuclear (Nuclear matrix)	Intergenic	X Chromosome inactivation.	In all placental mammals, with varying exonic and sequential conservation.	Its disruption can lead to cancer.	98, 99
*MEG3* (Figure 5B)	Nuclear	Intergenic	Acts as a tumour suppressor by stimulating p53. Regulates TGF‐β pathway genes. RNA–RNA interactions.	Tertiary structure conservation across most placental mammals. Unreliable detection across marsupials.	Associated with Huntington's disease and diabetic retinopathy. Tumour suppressor.	100, 101, 102
*linc‐RoR* (Figure 3C)	Cytoplasmic	Intergenic	miRNA sponge, role in maintenance of pluripotency.	No evidence	Promotes oncogenesis in human Esophageal Squamous Cell Carcinoma.	63, 64
*lincRNA‐p21* (Figure 6E)	Nuclear	Intergenic	Interacts with hnRNPK; Repressor of p53‐dependent transcriptional responses. Highly enriched in exosomes	Alu sequence elements conserved in mouse	Oncogenic role in prostate cancer.	48, 103, 104
*HOTAIR*	Nuclear	Anti‐sense	Hox gene expression regulation during development	Strong sequence conservation within primates, but poor across mammals. Evidence of structural conservation.	Oncogenic role in cancers including breast, gastric, colorectal and cervical.	105, 106, 107, 108
*yylncT* (Figure 3C)	Nuclear	Bi‐directional	Required for correct lineage specification during development	A syntenic transcript was detected in E6.5 mouse embryos	Protects development from aberrant *de novo* methylation.	62
*HOTTIP* (Figure 5C)	Nuclear	Anti‐sense	Participates in the spatial regulation of Hox gene expression during development	Conserved in mice and humans	Lung cancers.	109, 110
*NBAT‐1*	Nuclear	Anti‐sense	Transcription regulation, miRNA sponge.	Some sequence conservation across mammals.	Significantly lower expression in high‐risk neuroblastoma tumours. Potential biomarker for neuroblastoma progression.	70
*LINK‐A/LINC01139* (Figure 7B)	Nuclear/Cytoplasmic	Intergenic	Lipid binding, kinase activation.	No evidence	Upregulated in cancer, correlated with poor prognosis in breast cancer.	73
*BC200/ BCYRN1* (Figure 7C)	Cytoplasmic	Intergenic	Translation initiation repression in dendrites (RNPs)	Conserved in primates, has an orthologue in rodents	Implicated in Alzheimer's disease.	75
*HTT‐AS* (Figure 7D)	Nuclear	Anti‐sense	Transcription regulation	No evidence	Associated with Huntington's disease.	79

Another lncRNA, LINC00261, exhibits a lower level of sequence conservation than CHASERR. It shares only some sequence homology between mammals and fish in the first exon (Table 1). However, LINC00261 is also syntenically homologous in a wide range of species, from humans to sea urchins 13. Found downstream of *FOXA2*, LINC00261 is downregulated in multiple cancers and its overexpression inhibits cancer cell invasion, proliferation, and migration 16. In many cases, despite a lack of detectable sequence homology, the act of transcription of these syntenic lncRNAs could affect the expression of the neighbouring gene, demonstrating a conserved position and *cis*‐regulatory function.

For the majority of conserved lncRNAs, only a moderate level of sequence identity is observed 17. This is demonstrated by *Cerox1*, an intergenic, bi‐directional lncRNA found in mice (Table 1). *Cerox1* regulates the abundance of the mitochondrial complex 1 transcript by acting as a miRNA decoy, therefore modulating its activity. Although *Cerox1* is conserved at the sequence level and is syntenically homologous with a human homologue, the level of conservation drops rapidly in more distant species. Across eutherian mammals, conservation is found only in the second exon of *Cerox1* 18.

Of interest, the regulation and tissue specificity of lncRNAs are conserved to a level comparable to mRNAs 13. Specifically, conservation of lncRNA promoters is as strong as that of protein‐coding gene promoters 19. This suggests that selective constraints are often acting at the transcriptional level. However, lincRNA transcription also evolves at a rapid rate; only 72% of human lincRNAs are also expressed in macaque, compared to 98% of human protein‐coding genes in all primates 19.

A further complication in the identification of conserved lncRNAs is their evolution; the origin of lncRNAs is for the most part unknown. They also evolve rapidly, with the majority of lncRNAs found to be lineage specific 13. Due to the low levels of sequence conservation, it is generally assumed that lncRNAs are unlikely to evolve *via* gene duplication, a common mechanism for protein‐coding genes. Potential routes include protein‐coding genes losing their original function *via* truncation and loss of coding capacity and becoming lncRNAs. This mechanism is thought to account for a proportion of conserved lncRNAs, including Xist 20. A further possibility is that non‐coding portions of the genome may associate with a promoter, become transcribed, and eventually gain function as an lncRNA.

## Subcellular localisation

If an lncRNA is functional, its location in the cell will be important in understanding this function. Initial high‐throughput studies have indicated that lncRNAs were predominantly enriched in the nucleus 7. However, an increasing number of cytoplasmic lncRNAs are now being unearthed 21. All lncRNAs are transcribed in the nucleus and so are present there at some level. An area of active study is to understand which lncRNAs make it out the of nucleus and why. Enrichment of a lncRNA in either the nucleus or the cytoplasm does not preclude it from operating in both compartments. In fact, it is possible for lncRNAs to move between the two compartments in response to signals (e.g. UCHL1‐AS 22).

### Nuclear retention of lncRNAs

**Figure 4:**
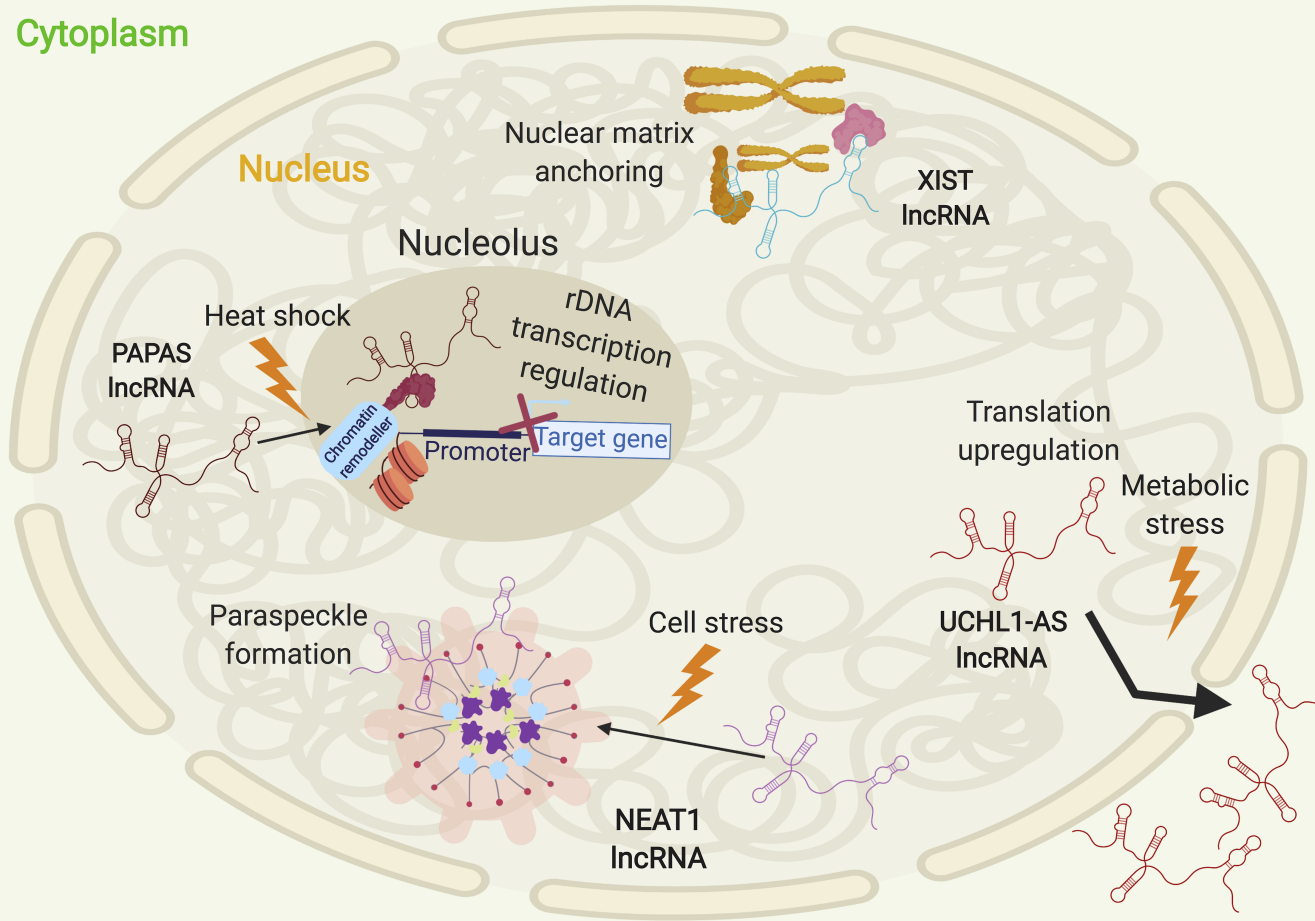
Localisation of nuclear lncRNAs to specific nuclear regions and their dynamic nature. lncRNAs generated in the nucleus can be anchored at specific locations (e.g. XIST) or dynamically shift their intra‐ and inter‐cellular localisation in response to environmental cues such as heat shock (e.g. PAPAS) or metabolic stress (e.g. UCHL1‐AS). Created using BioRender.

Many lncRNAs are enriched in the nucleus and a considerable number have been found to function specifically in the nucleus. Within the nucleus, many lncRNAs are specifically localised to nuclear sub‐compartments (Figure 4, Table 1), which argues against them being transcriptional by‐products. In fact, lncRNAs have been found to occupy the nucleolus (PAPAS), nuclear matrix (XIST), nuclear speckles (MALAT1), and nuclear paraspeckles (NEAT1) (Figure 4). Targeting of lncRNAs to such specific locations can be the result of nuclear localisation signals within the lncRNA itself (e.g. BORG and MALAT1 23, 24).

Analysis of nuclear lncRNAs sequences has identified specific motifs enriched in lncRNA correlating with nuclear fate 25, 26. For instance, a 57 nt motif is repeated 18 times in Xist, and a 15 nt C‐rich element is found in 21 different lncRNAs, including MALAT1. Essentially, the presence of several of these motifs in an lncRNA is more likely to facilitate its nuclear retention. Several lncRNA sequence elements, which have evolved from transposable elements (TEs), have also been characterised as sufficient for nuclear localisation (e.g. L2B, MIRb, and MIRc TE 27). Similarly, a less‐stringent analysis revealed the involvement of Alu repeat motifs in nuclear retention of lncRNAs 28. The location and number of these elements as well as flanking sequences have been shown to contribute to the strength of nuclear enrichment.

RNA splicing is important to the export of mRNAs from the nucleus. Unsurprisingly, inefficient splicing of lncRNAs has been linked to their nuclear localisation (e.g. A‐ROD lncRNA), and intron retention contributes to restricting of lncRNAs to the nucleus 29. Weak interactions of lncRNAs with splicing factors (e.g. MALAT‐1 with SRm160 23) is also thought to contribute to nuclear retention. The U1 snRNP complex keeps lncRNAs in the nucleus in two ways 30, 31. LncRNA exons and introns contain U1 recognition sites, which U1 snRNP (small nuclear ribonucleoprotein) and associated factors have been shown to be recruited to and antagonise cytoplasmic export 31. These sequences have been found in nuclear retention elements. It has also been speculated that U1 spliceosome association with U1 recognition sites then contributes to inefficient splicing and remaining bound to chromatin. As a result, polyadenylation of these transcripts is impeded, allowing for lncRNA turnover 30.

Those lncRNAs that do not interact with the mRNA export machinery (e.g. Aly/REF) are more likely to be enriched in the nucleus 32. Xist lncRNA is retained within the nucleus partly as a result of its weak interactions with RNA export proteins such as Nxf1 33. Export of some lncRNAs with limited splicing (e.g. NORAD) is facilitated by splicing‐independent export machinery such as TPR 34.

LncRNAs can be tethered to the nuclear matrix or nuclear (para)speckles (membrane‐less organelles) by adapter proteins. For example, CIZ1 and hnRNPU, which can interact with chromatin, facilitate Xist lncRNA anchoring to the nuclear periphery (Figure 4) 35, 36 and similarly with Bloodlinc 37.

### Function of nuclear lncRNAs

**Figure 5:**
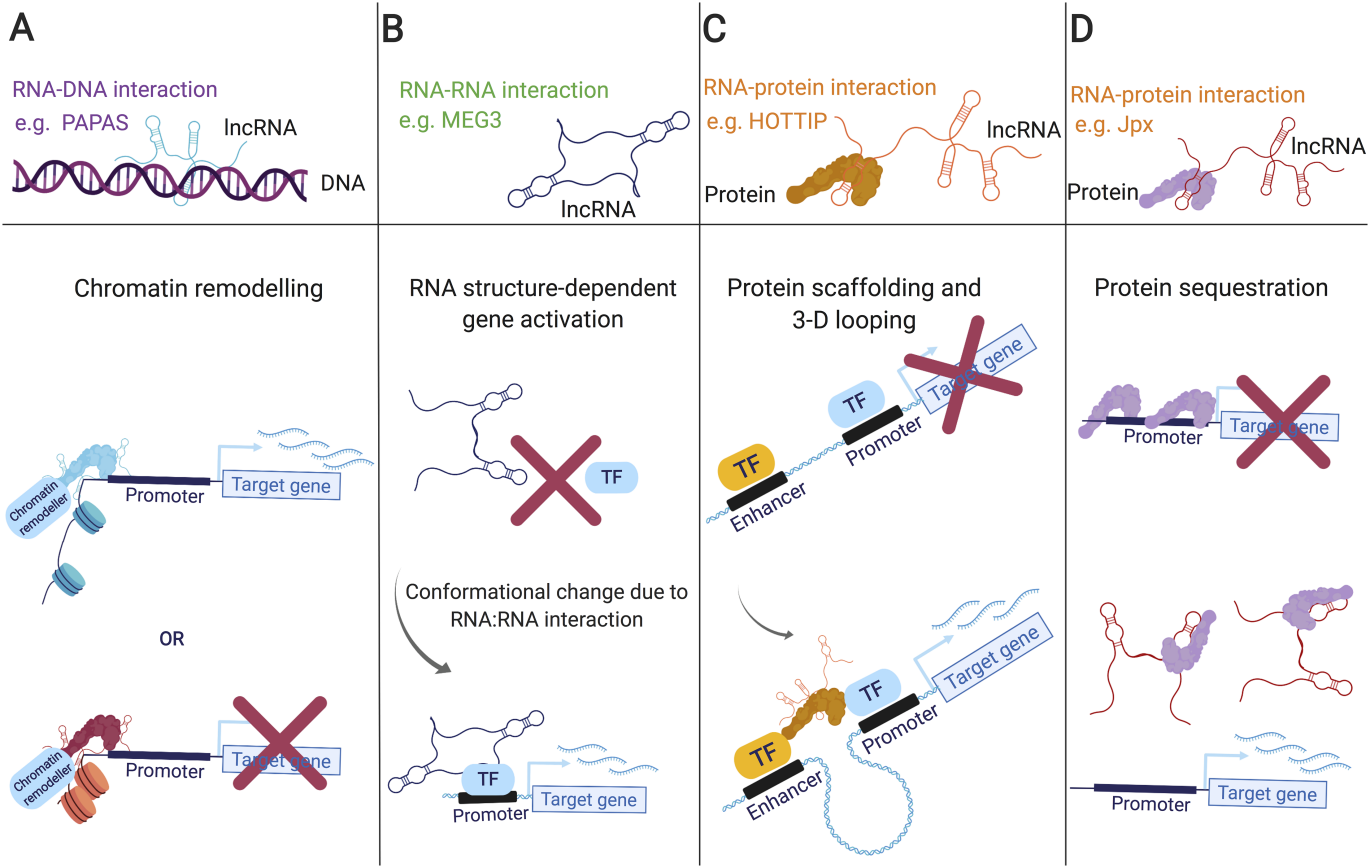
Molecular functions of nuclear lncRNAs. (A) lncRNAs can guide chromatin remodelling complexes to transcription sites, which can either deposit active or repressive chromatin marks (e.g. Xist). (B) lncRNA:RNA interactions can cause a shift in tertiary structure, activating transcription factors which regulate gene expression (e.g. MEG3). (C) By bridging protein interactions or scaffolding the assembly of multi‐protein complexes, lncRNAs can facilitate enhancer and promoter element interactions, critical for gene activation (e.g. HOTTIP). (D) IncRNAs bind specific proteins with high affinity to titrate proteins away from typical occupancy sites, impacting gene activity and/or 3‐D genome compaction (e.g. Jpx). Created using BioRender.

As mentioned previously, lncRNAs can interact with DNA, RNA, and proteins. Nuclear lncRNAs tend to engage with DNA and protein (Figure 3) to regulate gene expression in a variety of ways (Figure 5). For instance, lncRNA PAPAS binds both DNA and protein to elicit its function. PAPAS interacts with the CHD4/NuRD (nucleosome remodelling and deacetylation) complex and can engage in RNA–DNA triplex formation to ‘scout’ for its DNA recognition element, effectively guiding chromatin remodellers to sites necessary for gene‐expression regulation 38 (Figure 5A). lncRNA MEG3 interacts with DNA to guide chromatin remodellers, interacts with itself (lncRNA:lncRNA) 39, and acts as a co‐activator of p53, partially regulating p53 target genes 40 (Figure 5B, C). Although lncRNA Jpx competes with DNA for CTCF protein docking, the action of titrating CTCF away from one of the two X chromosomes in mice contributes to X chromosome inactivation (Figure 5D).

### Cytoplasmic lncRNAs

Export of lncRNAs to the cytoplasm is usually indicative of a specific function, given that some level of activity is required. A recent study in the human chronic myelogenous leukaemia cell line (K562) revealed that 54% of expressed lncRNAs are detected in the cytoplasm 27. The majority of 5′‐capped, spliced, and polyadenylated lncRNAs ‘get the green light’ to reach the cytoplasm. Methylation of adenosine (N^6^‐Methyladenosine, m^6^A) in lncRNAs, like mRNAs, stimulates their export to the cytoplasm. For mRNAs, this is mediated by YTHDC1, which binds to the modified base and helps recruit nuclear export factors (41 and references therein). Although there is no direct evidence, this is probably also the case for lncRNAs. Although the presence of some specific TEs promote nuclear lncRNA retention, some classes of TEs (e.g. the endogenous retrovirus class ERVL‐MaLR) are enriched in cytoplasmic lncRNAs 27. This suggests that there are specific sequence determinants of lncRNAs being localised to the cytoplasm. Of interest, lncRNA localisation to the cytoplasm can be affected by external cues and, therefore, can change. For example, UCHL1‐AS shifts from the nucleus to the cytoplasm upon stress induction with rapamycin in mouse dopaminergic MN9D cells 22.

**Figure 6:**
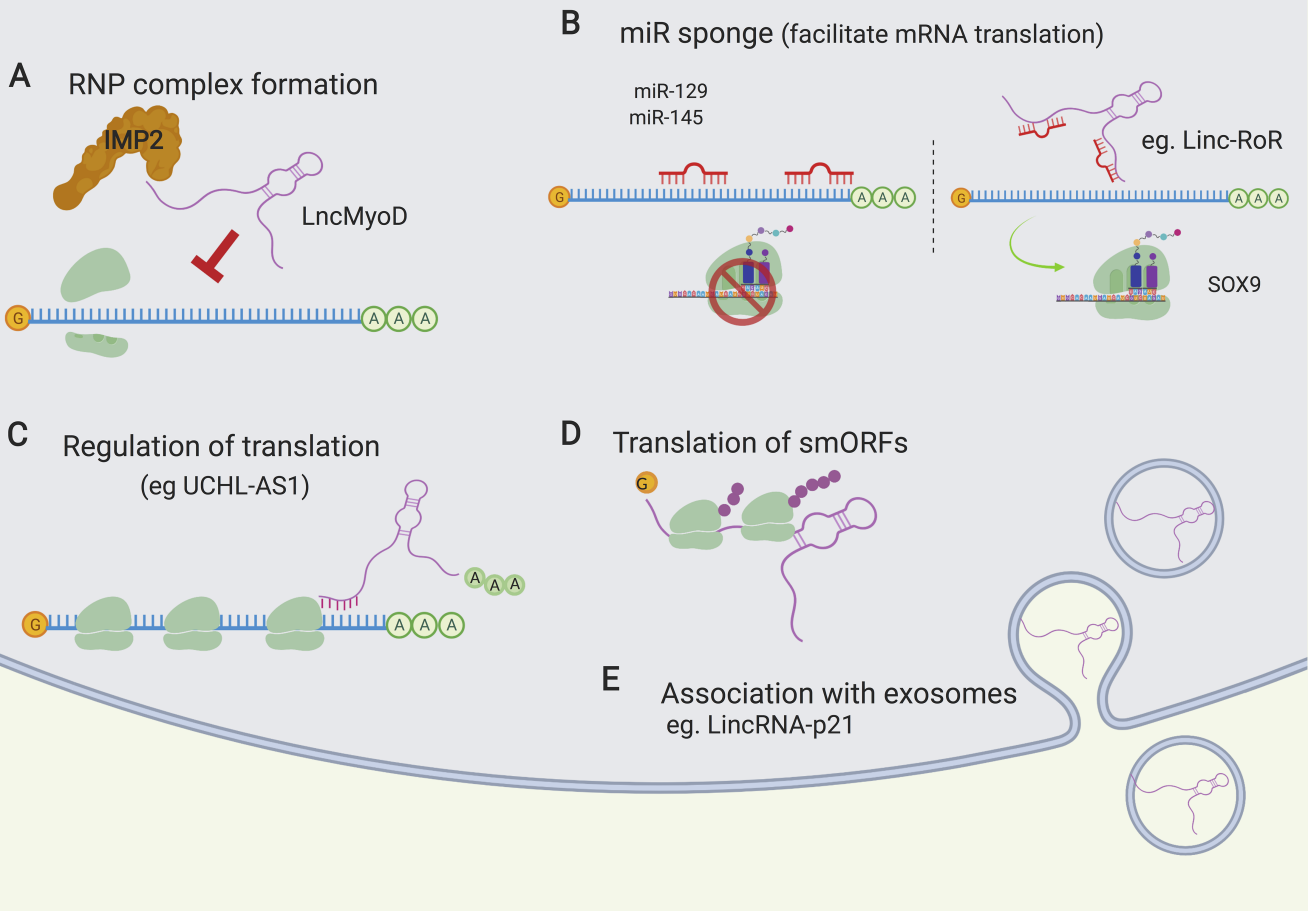
Molecular functions of cytoplasmic lncRNAs. (A) lncRNAs interact with proteins and/or mRNAs to form RNP complexes, which regulate post‐transcriptional gene regulation. (B) lncRNAs act as molecular sponges for miRNAs, thus stabilising and protecting mRNAs from degradation. (C) lncRNAs associate with the translation machinery and regulate the translation of mRNAs. (D) lncRNAs can be actively engaged by translating ribosomes. (E) lncRNAs have been found in extracellular vesicles. Created using BioRender.

As in the nucleus, cytoplasmic lncRNAs can form ribonucleoprotein complexes by binding mRNAs and proteins, or by competing with mRNAs for binding to a specific protein (Figure 6A). lnc MyoD, which plays a key role in the regulation of myogenesis, is one such an example. It interacts strongly with IGF2 mRNA‐binding proteins (IMPs), and negatively regulates IMP2‐mediated translation of proliferation genes such as N‐Ras and c‐Myc by antagonising those mRNAs for IMP binding, thus promoting cell cycle exit and differentiation 42.

Some lncRNAs harbour miRNA‐binding sites and by having multiple sites can act as ‘molecular sponges’, sequestering miRNAs and therefore protecting mRNAs from miRNA‐mediated degradation. These lncRNAs are termed competing endogenous RNAs (ceRNAs) 43. One such molecular sponge is the lncRNA regulator of reprogramming (linc‐RoR), which interacts with miR‐145 (Figure 6B).

As already mentioned, many lncRNAs are capped and polyadenylated 7, 17. If these lncRNAs are present in the cytoplasm, they can interact with ribosomes (Figure 6C) and be translated (Figure 6D). In fact, a number of studies in human, mouse, fly, and yeast have detected lncRNAs in ribosome‐bound complexes by ribosome profiling 44, 45, 46. This RNA‐Seq–based method, which detects translation events, has revealed that small open reading frames (smORFs, <100 aa) present in some lncRNAs are actually translated. Given that many cytoplasmic lncRNAs possess the same molecular characteristics as mRNAs, this should not be that surprising. Should these translated lncRNAs be re‐classified as mRNAs? Because lncRNAs by their very nature lack long open reading frames, these translation events generally can produce only small peptides. These lncRNA–ribosome interactions remain controversial, as they could represent non‐specific interactions rather than bonafide translation events.

In addition to lncRNAs being actively engaged by ribosomes, lncRNAs can also associate with mRNAs during translation 22 (Figure 6D). Several lncRNAs are associated with large translation complexes (polysomes), but they themselves are not translated. These polysome‐associated lncRNAs tend to exhibit widespread expression patterns across different human tissues. They are also more likely to be enriched in the cytoplasm compared to free cytoplasmic lncRNAs 27. This suggests that these polysome‐associated lncRNAs are more likely to be functional than those present just in the cytosol.

Cytoplasmic lncRNAs may localise to specific organelles or cytoplasmic structures. Notably, some cytoplasmic lncRNAs are encoded by mitochondrial DNA, and are therefore found and operate in the mitochondria 18, 47. lncRNAs participate in the formation of P‐bodies and extracellular vesicles (EVs), the latter of which are secreted from one cell and received by another, acting as cellular messengers (Figure 5E). It has been suggested that lncRNA molecules with relatively low expression levels (e.g. lincRNA‐p21, HOTAIR) are highly enriched in exosomes (Table 1) 48. The level of secretion of lncRNAs is critical for cell homeostasis, as it has been shown that lncRNA exosome levels reflect the cellular response to DNA damage 48. LincRNA‐p21 transcript levels in exosomes isolated from urine samples of prostate cancer patients appear to be significantly elevated compared to those of patients with benign prostatic hyperplasia 49. Therefore, LincRNA‐p21 could serve as a biomarker for the differential diagnosis between prostate cancer and benign prostatic hyperplasia.

## LncRNA roles in development and differentiation

LncRNAs have been found not only to be spatially restricted but also temporally, that is, at certain stages in development. Specific expression of a lncRNA during development suggests that it may have an important function at that time. Several such lncRNAs have been characterised. Probably the most well‐known lncRNA is XIST (Table 1), a nuclear‐retained lncRNA that is 5′ capped, polyadenylated, and alternatively spliced (~19.2 kb in human) 50. XIST is important during early development, functioning in X chromosome inactivation (XCI) as part of dosage compensation. Mutations in mouse Xist result in an inability to undergo XCI and embryonic lethality 51, 52.

To ensure an equal dosage of X‐linked genes between females and males, one of a female's two X chromosomes is inactivated. Post‐differentiation, Xist is expressed specifically in females. In humans, XIST is lowly expressed from day 4 shortly after zygote formation, increasing at days 6 and 7 post‐fertilisation with random XCI taking place on day 7 53. The precise timing of XCI differs in diverse placental mammals 54, as does Xist's precise temporal expression. Studies in human and mouse stem cells suggest that Xist recruits protein partners, forming several lncRNA–protein complexes and covering one X chromosome to inactivate it. Xist recruits SPEN to initiate gene silencing 55, 56, 57, 58, and then, *via* hnRNPK, PRC1, and PRC2 complexes, to deposit repressive chromatin marks to establish and maintain gene silencing (Figure 5A, 59). hnRNPU and CIZ1 tether Xist to the nuclear periphery (Figure 4) 35, 36, 60, 61.

The establishment of the three germ layers (lineage specification) from the embryonic epiblast is a critical point in development. lncRNAs have been found to control the direction of stem cell differentiation and hence contribute to cell identity decisions. For instance, yylncT lncRNA has been detected in the nucleus of day 2 human embryonic stem cells (hESCs) following mesoderm specification 62. Disruption of yylncT transcription or its depletion in differentiating hESCs leads to decreased levels of the transcription factor Brachyury, a master regulator of mesoderm specification, resulting in increased apoptosis and downregulation of key mesoderm driver genes. yylncT lncRNA safeguards both its own and the Brachyury locus from genome‐wide methylation in response to differentiation signals 62.

Cytoplasmic lncRNAs are also important in pluripotency and differentiation by interacting with key pluripotency factors. Linc‐RoR has been shown to crosstalk with some of these factors, namely OCT4, SOX2, and NANOG, specifically in self‐renewing hESCs. In fact, linc‐RoR levels are markedly reduced in differentiated hESCs, implying a key role in pluripotency 63. Linc‐RoR can act as a ceRNA to sequester miR‐145, which negatively modulates *OCT4*, *SOX2*, and *NANOG* mRNA levels (Figure 6B). Essentially, linc‐RoR can block exit from pluripotency *via* an miR‐145‐mediated *OCT4* downregulation pathway in the cytoplasm. Linc‐RoR also deregulates the pluripotency transcription factor *SOX9* through competition with miR‐15b, miR‐33a, miR‐129, miR‐145, and miR‐206, in human oesophageal squamous cell carcinoma cells and patients' samples, resulting in Sox9 stabilisation and promotion of oncogenesis 64. Upregulation of linc‐RoR has been linked to the increased occurrence of cancers such as triple negative breast cancer 65, and endometrial 66, nasopharyngeal 67, and liver cancers 68.

Determination of the anterior–posterior axis is mediated by the coordinated expression of Hox genes. Along with *Hox* protein‐coding genes, Hox lncRNAs have also been shown to participate in regulating this process. HOTTIP is a Hox lncRNA that is functionally conserved across developing mice, chick embryos, and humans. It modulates precise spatial and temporal expression patterns of *Hox* genes, ensuring proper embryonic development. Chick embryos with reduced HOTTIP expression exhibit defects in limb development 69. HOTTIP lncRNA can recruit histone methyltransferase complexes to deposit gene activation chromatin marks (i.e. H3K4me3) on the 5′ *Hox‐A* locus (Figure 5). These examples illustrate how important lncRNAs are to development, acting as gatekeepers for cell viability or cell fate specification.

## LncRNAs in cancer

It is evident that lncRNAs play decisive regulatory roles at crucial checkpoints between proliferation and differentiation. When this regulation is disrupted, disease is the inevitable consequence. Although early work uncovered lncRNAs associated with cancer, there are now functionally characterised lncRNAs whose contribution to cancer progression and phenotype has been elucidated at a more mechanistic level.

NBAT‐1 is a well‐studied lncRNA with a defined role in cancer, the mechanism of action for which has recently been elucidated. Like many lncRNAs, NBAT‐1 exhibits tissue specificity and is expressed mainly in the brain, breast, and ovary. Notably, some regions of NBAT‐1 have substantial sequence conservation across mammals, suggesting an evolutionarily conserved function 70. Cancer patients with high NBAT‐1 expression have been associated with good prognosis, whereas patients with low NBAT‐1 expression have been associated with poor prognosis 70. NBAT‐1 contains a high‐risk associated single nucleotide polymorphism (SNP) within its intron, which contributes to regulating its expression levels (A/A genotype higher NBAT‐1 expression, G/G genotype lower NBAT‐1 expression). In high‐risk patients, DNA methylation functions to inactivate the NBAT‐1 promoter, leading to downregulation of expression and consequently to cell proliferation and invasion. The SNP and NBAT‐1 promoter may together be involved in higher order regulatory interactions.

**Figure 7:**
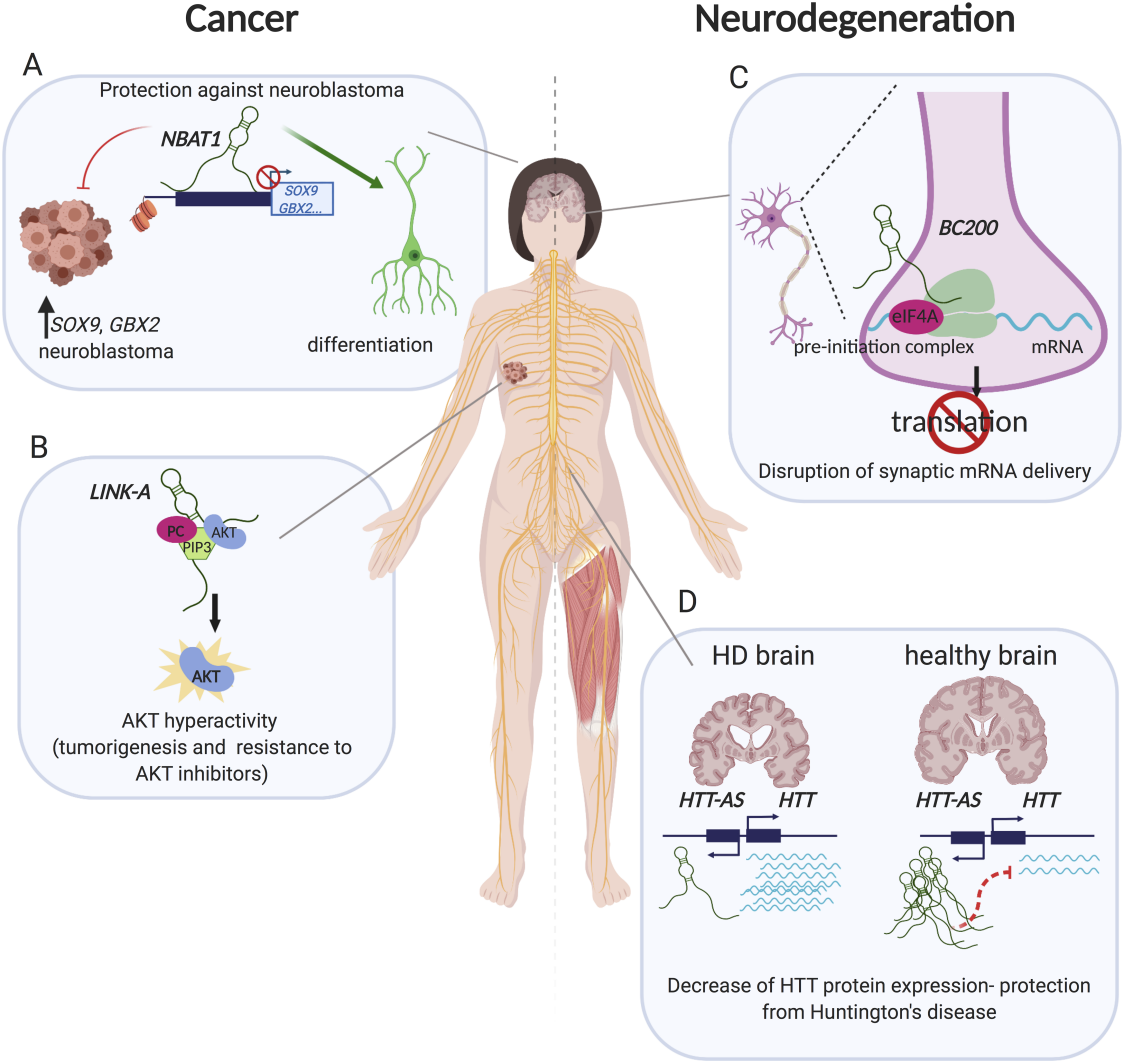
lncRNAs can promote or protect from cancer and neurodegeneration. (A) NBAT1‐lncRNA promotes neuronal differentiation and prevents neuroblastoma. (B) LINK‐A interacts with PIP3, PC, and AKT, resulting in AKT hyperactivity and drug resistance in breast cancer. (C) BC200 disrupts mRNA delivery and translation at the synapse and leads to neurodegeneration. (D) HTT‐AS controls HTT expression levels. Downregulation of HTT‐AS has been linked to the occurrence of Huntington's disease. Created using BioRender.

Suppression of NBAT‐1 by knockdown in the SH‐SY5Y human neuroblastoma cell line results in increased cancer cell viability and invasiveness, whereas overexpression leads to decreased cell proliferation and invasion. Furthermore, mouse xenografts developed from NBAT‐1–depleted cells showed a substantial increase in growth rate. RNA‐seq of NBAT‐1 knockdown in SH‐SY5Y identified several genes associated with cell proliferation and migration that are regulated by NBAT‐1. Among these are *VCAN*, *SOX9*, and *OSMR*, which contribute to cancer progression 71, 72. NBAT‐1 lncRNA interacts with the PRC2 complex member EZH2 to suppress target genes implicated in cell proliferation and cell migration *via* chromatin level regulation 70. Taken together, these findings suggest that NBAT‐1 is a ‘protector’ against neuroblastoma (Figure 7A).

On the other hand, some lncRNAs that are misregulated in cancer act to promote tumour growth and resistance to chemotherapy. One such example is LINK‐A (LINC01139; long intergenic non‐coding RNA for kinase activation). The LINK‐A locus is amplified in multiple cancer types, and high expression levels of LINK‐A are correlated with poor prognosis in breast cancer patients (Figure 7B) 73. Of interest, LINK‐A is cytoplasmic in adenocarcinoma lung cells and SK.N.SH cells, but nuclear in HeLa and NHEK cells 21. This suggests that it shuttles between the nucleus and cytoplasm. LINK‐A belongs to the category of lipid‐binding lncRNAs, and specifically binds to phosphatidylcholine, AKT, and PIP3, resulting in AKT hyperactivation that leads to tumorigenesis and resistance to AKT inhibitors 73. LINK‐A interacts with PIP3 through a 60 nt RNA oligonucleotide motif (nt 1081–1140), containing a stem‐loop structure essential for this interaction. Furthermore, LINK‐A–PIP3 interaction confers resistance to anti‐tumour drugs such as perifosine and MK2206.

Mechanisms of action at the molecular level and association with cancer have been studied for a substantial number of lncRNAs so far. There seem to be two distinct patterns of action: they can either inhibit cancer cell proliferation and invasion, or promote tumour growth and metastasis.

## LncRNAs and neurodegeneration

About 40% of human lncRNAs (equivalent to 4000‐20 000 lncRNA genes) are specifically expressed in the brain 7, where they display an exquisite spatiotemporal expression profile as well as cellular localisation variability 74. lncRNAs are involved in key regulatory mechanisms during various stages of neurogenesis and synaptic plasticity. Therefore, it is not surprising that the misregulation of several lncRNAs contributes to the development of neurodegenerative diseases.

Primate‐specific brain cytoplasmic RNA BC200 (or BCYRN1) and its rodent orthologue (BC1) are cytoplasmic anti‐sense lncRNAs highly expressed in the brain (Table 1) and have been implicated in Alzheimer's disease (AD). They both regulate mRNA translation, selectively target to somatodendritic domains of human neurons, and contribute to the maintenance of long‐term synaptic plasticity 75. Specifically, they have been reported to repress translation initiation in dendrites 76 (Figure 7C). BC200 exhibits a diffuse localisation in dendritic domains of neuropils and its levels are reduced during ageing. In AD, BC200 is upregulated in specific areas of the neocortex, where it forms characteristic clusters mislocalised in cell soma rather than in the synapse 75. It seems to disrupt mRNA localisation to the synapse, which is required for proper synapse function 77. Levels of BC200 across different parts of the brain correlate with the severity of the disease in these brain regions 75, further suggesting an important role.

The recently characterised lncRNA UCHL1‐AS has been associated with Parkinson's disease and exhibits a dynamic subcellular localisation (Table 1). UCHL1‐AS is in the anti‐sense orientation to *UCHL1*, which produces a neuron‐restricted protein, essential for development. UCHL1‐AS is conserved between mouse and human. It is highly expressed in the murine ventral midbrain and in mouse MN9D dopaminergic cells 22. Normally UCHL1‐AS accumulates in the nucleus of dopaminergic neurons. Upon cellular stress induced by rapamycin (inhibitor of cap‐dependent translation), UCHL1‐AS translocates to the cytoplasm where it promotes translation of *UCHL1* mRNA by increasing its association with heavy polysomes 22. UCHL1‐AS is downregulated in a mouse neurochemical model of Parkinson's disease 78.

Huntington's disease (HD) is caused by a CAG trinucleotide repeat expansion in exon 1 of the huntingtin gene, *HTT* 79. Several well‐studied lncRNAs, such as NEAT1 and MEG3, have been associated with HD 80. A recently discovered anti‐sense lncRNA, HTT‐AS, regulates the expression of *HTT* 79. HTT‐AS is 5′ capped, polyadenylated, and can be alternatively spliced 79. HTT‐AS levels are reduced by 50% in HD brains compared to controls, and it negatively regulates *HTT* expression. Knockdown of HTT‐AS leads to a 20% increase of *HTT*, whereas overexpression of HTT‐AS decreases endogenous *HTT* expression by 25% 79 (Figure 7D).

These three lncRNAs highlight how lncRNA misregulation is implicated in neurodegeneration. Each of them acts at a different level of gene expression regulation, a key feature of lncRNAs in general.

## LncRNAs as biomarkers and therapeutic targets

Despite the small number of functionally characterised lncRNAs, their potential as biomarkers or therapeutic agents is quickly being realised. Following the huge progress in elucidating the mechanisms of action and the association of lncRNAs with disease, the scientific community is now keen to investigate the possibility of using lncRNAs as prognostic or diagnostic biomarkers. This approach is based on the initial observations that the levels of certain lncRNAs in blood samples correlates with the occurrence of a disease. In a preclinical setting, lncRNAs have been suggested as potential diagnostic or prognostic biomarkers affecting a variety of tissues including the heart (MALAT1), reproductive system (H19), muscle (linc‐MD1), and several cancer types (MALAT1, H19, MEG3, HOTAIR) (reviewed in 81, 82).

LncRNAs are ideal biomarker candidates because of their high tissue specificity. This may enable the detection of metastasis, or perhaps help alleviate the complexity imposed by cancer heterogeneity 81. As mentioned, lncRNAs can be encapsulated in EVs and so have the potential to end up in the blood stream, also making them ideal biomarkers 83. The temporal restriction of some lncRNAs could add another layer to biomarker specificity, or even allow for the tracking of disease progression. Several lncRNAs have already been explored for these reasons, but none have reached clinical trials yet.

GAS5 lncRNA is being currently studied at a pre‐clinical level as a potential biomarker for type 2 diabetes (T2D) and coronary artery disease (CAD). GAS5 is readily detected in human serum and its levels are correlated to the prevalence/onset/appearance of T2D. Specifically, GAS5 is significantly downregulated in diabetic patients compared to healthy controls 84, and significantly lower in patients with CAD compared to patients with diabetes mellitus 85.

NBAT1 also has the potential to serve as a prognostic/diagnostic marker for different types of cancer. Although it is not being tested in clinical trials yet, there is substantial pre‐clinical evidence from patient samples. It is differentially expressed in transcriptomic profiling of 15 neuroblastomas of different types 70. NBAT‐1 can be utilised for differential diagnosis, because patients with high NBAT‐1 are associated with good prognosis and *vice versa* 70. NBAT‐1 levels are considerably lower in bladder cancer samples compared to healthy controls 86.

SNGH1 could represent a novel lncRNA biomarker of tumorigenesis for a number of tissue biotypes including brain, breast, lung, liver, and ovary 87. SNGH1 lncRNA can be detected at a higher level in tissues from patients with colorectal carcinoma compared to healthy individuals. It is important to note that high levels correlate with tumour malignancy, reflected in poorer patient survival compared to those with lower SNGH1 levels. In cell lines, enhanced apoptosis and diminished cell proliferation were observed when SNGH1 was decreased, suggesting it might represent a therapeutic target as well as acting as a potential biomarker.

RNA therapeutics is a newly emerging but rapidly expanding field. Most approaches so far have included targeting an offending RNA molecule with small molecule drugs or using anti‐sense oligos to specifically interfere with RNA function. Using RNA molecules as drugs is also being explored by industry. Targeting the biogenesis of lncRNAs is currently being pursued in cancer cell lines 88, 89. This approach has been employed previously with miRNAs (reviewed in 90).

LncRNAs themselves could act as therapeutic molecules. One approach has been described whereby lncRNAs harbouring tuneable sequence elements (known as SINE‐UPs) can be tailored to target mRNAs and specifically increase their translation. This highly customisable approach could be adapted for any disease where physiological protein levels are disrupted in a cell, such as Friedreich's ataxia 91. These elements on specific lncRNAs can also be multiplexed to target a series of transcripts in parallel, achieving multiple target regulation.

Therapeutic strategies to modulate lncRNA activity have now made it to clinical trials. Abivax has developed a small molecule that has entered phase 2 clinical trials for ulcerative colitis and moderate to severe stage rheumatoid arthritis patients 92, 93, 94. It increases splicing of lncRNA 0599‐205, upregulating production of miR‐124 due to this lncRNA harbouring one of three miR‐124 loci in the genome 95. miR‐124 has been shown previously to reduce pro‐inflammatory cytokines and also regulate innate and adaptive immunity. Thus, by increasing the levels of spliced lncRNA 0599‐205, miR‐124 production is increased and inflammation in arthritis is reduced 95.

It is too early to speculate on whether lncRNA therapeutics will be widely applicable. Nonetheless, there is growing interest in developing and adapting lncRNAs from a preclinical to a clinical setting. The more we understand the function of lncRNAs and how they contribute to disease, the more they can be exploited as therapeutic targets.

## Concluding remarks

There are more than 16 000 lncRNA genes in the human genome, but we currently understand the function of only ~50. Our improved appreciation of lncRNAs is changing the way we think about our genomes and genetic diseases. The simplistic view that lncRNAs remain in the nucleus to regulate transcription has been expanded in the last 5 years to appreciate the various ways both cytoplasmic and nuclear lncRNAs can act. In fact, their movement between cell compartments may be dynamic, and their roles can vary depending on their cellular location.

The low abundance and highly specific expression patterns of lncRNAs have made studying them difficult. Function has also been incorrectly assigned to lncRNAs based on CRISPR experiments, and now siRNAs are the preferred method to downregulate lncRNAs without creating genomic alterations, which may be lncRNA independent. Not all lncRNAs possess a function and determining which do and which do not is a substantial body of research. Conservation analysis will contribute to this understanding and identify model systems in which research can be performed. Those lncRNAs whose function has been determined have been found to contribute to development, neural function, and cancer (Figure 7).

We have also made significant progress from simple disease association to mechanistic insights to reveal how lncRNAs contribute to disease phenotypes. This in turn allows lncRNAs to be used as therapeutic targets as well as biomarkers. Given the number of human lncRNAs, they provide a vast screening space whose potential has yet to be fully realised. Further work to precisely map mutations to genetic diseases will no doubt uncover many more lncRNAs whose disruption gives rise to disease. Studies such as the 100 000 Genomes Project will be key to uncovering precise links with pathology.

## Author contributions statement

IT, KD, IB, and JA were all involved in writing the review and had final approval of the submitted and published versions.

## Glossary of terms

**Homology:** similarity of a nucleotide or amino acid sequence between species. **In *cis*:** function of a transcript at or very close to the site of its transcription. **In *trans*:** function of a transcript away from the site of its transcription. **Orthologues:** homologous genes found in different species following a speciation event, for which gene sequences and main function are conserved. **Ribonucleoprotein complexes (RNPs**): complexes formed by RNAs interacting with RNA‐binding proteins. **Ribosome profiling/Ribo‐Seq:** high‐throughput sequencing of small RNA fragments‐protected by ribosomes. **Synteny:** physical co‐localisation of genetic loci on the same chromosome within an individual or species. **ENCODE project:** ENCyclopedia Of DNA Elements project is a public research project aiming to identify functional elements in the human genome. **m** ^**7**^ **G cap:** methyl guanosine nucleotide added at 5′ end of mRNA to protect it from degradation and facilitate ribosome binding during translation. **CNS:** central nervous system.

## Abbreviations

AD, Alzheimer's disease; CAD, coronary artery disease; ceRNA, competing endogenous RNA; EV, extracellular vesicle; HD, Huntington's disease; hESC, human embryonic stem cell; IMP, IGF2 mRNA‐binding protein; lincRNA, long intergenic non‐coding RNA; lncRNA, long non‐coding RNA; m^6^A, N^6^‐Methyladenosine; nt, nucleotide; RBP, RNA‐binding protein; smORF, small open reading frame; snRNP, small nuclear ribonucleoprotein; SNP, single nucleotide polymorphism; T2D, type 2 diabetes; TE, transposable element; UTR, untranslated region; XCI, X chromosome inactivation.
